# Electrochemical Control of the Ultrafast Lattice Response of a Layered Semimetal

**DOI:** 10.1002/advs.202411344

**Published:** 2024-12-16

**Authors:** Felipe A. de Quesada, Philipp K. Muscher, Eliana S. Krakovsky, Aditya Sood, Andrey D. Poletayev, Edbert J. Sie, Clara M. Nyby, Sara J. Irvine, Marc E. Zajac, Duan Luo, Xiaozhe Shen, Matthias C. Hoffmann, Patrick L. Kramer, R. Joel England, Alexander H. Reid, Stephen P. Weathersby, Leora E. Dresselhaus‐Marais, Daniel A. Rehn, William C. Chueh, Aaron M. Lindenberg

**Affiliations:** ^1^ Department of Materials Science and Engineering Stanford University Stanford CA 94305 USA; ^2^ Stanford Institute for Materials and Energy Sciences SLAC National Accelerator Laboratory Menlo Park CA 94025 USA; ^3^ Computational Physics Division Los Alamos National Laboratory Los Alamos NM 87545 USA; ^4^ Department of Mechanical and Aerospace Engineering Princeton University Princeton NJ 08544 USA; ^5^ Princeton Materials Institute Princeton University Princeton NJ 08540 USA; ^6^ Department of Materials University of Oxford Oxford OX1 3PH UK; ^7^ SLAC National Accelerator Laboratory Menlo Park CA 94025 USA; ^8^ Stanford PULSE Institute SLAC National Accelerator Laboratory Menlo Park CA 94025 USA

**Keywords:** electrochemical lithiation, in‐operando, phase control, ultrafast electron diffraction, WTe_2_

## Abstract

The unique layer‐stacking in two‐dimensional (2D) van der Waals materials facilitates the formation of nearly degenerate phases of matter and opens novel routes for the design of low‐power, reconfigurable functional materials. Electrochemical ion intercalation between stacked layers offers a promising approach to stabilize bulk metastable phases and to explore the effects of extreme carrier doping and strain. However, in situ characterization methods to study the structural evolution and dynamical functional properties of these intercalated materials remains limited. Here a novel experimental platform is presented capable of simultaneously performing electrochemical lithium‐ion intercalation and multimodal ultrafast characterization of the lattice using both electron diffraction and nonlinear optical techniques. Using the layered semimetal WTe_2_ as a model system, the interlayer shear phonon mode that modulates stacking between 2Dlayers is probed, showing that small amounts of lithiation enhance the amplitude and lifetime of the phonon, contrary to expectations. This results from the dynamically fluctuating and anharmonic structure between nearly degenerate phases at room temperature, which can be stabilized by electronic carriers accompanying the inserted lithium ions. At high lithiation, the T_d_’ structure emerges and quenches the phonon response. This work defines new approaches for using electrochemistry to engineer the dynamic structure of 2D materials.

## Graphical abstract

A novel experimental platform is presented, capable of performing electrochemical lithiation of van der Waals materials and allowing for simultaneous multimodal characterization of the material using both ultrafast electron diffraction and nonlinear optical methods. By monitoring the behavior of the interlayer shear phonon in WTe_2_, light lithiation is found to stabilize the otherwise dynamically fluctuating and anharmonic structure at room‐temperature.

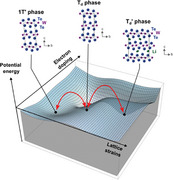

## 1 Introduction

**Figure 1:**
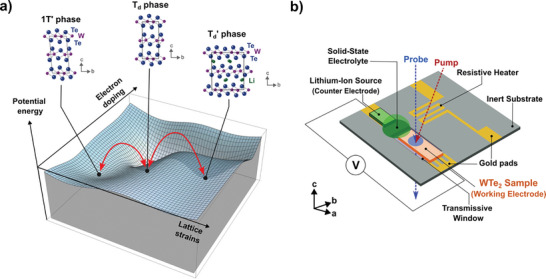
Phase control of WTe_2_ using electrochemical lithium‐ion intercalation. a) Qualitative illustration of the potential energy landscape of WTe_2_ featuring its T_d_, 1T’, and T_d_’ phases. The insets above the energy landscape show the unit cell of each structural phase projected onto the *bc*‐plane. b) Schematic representation of the experimental, all‐solid‐state intercalation platform.

Two‐dimensional (2D) layered materials, such as the transition metal dichalcogenides, feature a unique layer‐stacking degree‐of‐freedom that originates from the weak van der Waals interlayer interactions that bind the constituent layers together.^[^ ^1^, ^2^, ^3^, ^4^ ^]^ As a result, these materials support the formation of extraordinarily tunable and nearly energetically degenerate phases of matter, which open novel routes for low‐energy, reconfigurable devices and create opportunities for applications including neuromorphic computing, ultrafast sensing elements, and nanoscale thermal actuators.^[^ ^5^, ^6^, ^7^, ^8^ ^]^ One prominent example of such a system is semimetallic tungsten ditelluride (WTe_2_).^[^ ^9^ ^]^ This ferroelectric and topologically non‐trivial material resides in the orthorhombic T_d_ phase at room‐temperature (space group *Pmn2_1_*), but can be switched into the metastable, non‐ferroelectric monoclinic 1T’ structure that restores out‐of‐plane inversion symmetry (space group *P2_1_/m*) (**Figure** **1**a).^[^ ^6^, ^10^, ^11^, ^12^, ^13^ ^]^ Importantly, theoretical calculations predict that the T_d_ and 1T’ phases are only separated by a 43 picometer shear displacement between adjacently stacked layers along the *b*‐axis of the crystal, which is associated with an energy barrier of a few meV per f.u., enabled by the weak van der Waals bond.^[^ ^6^, ^10^, ^12^, ^14^ ^]^ This barrier between T_d_ and 1T’, which is small compared to *k_B_T* at room temperature, implies a dynamically fluctuating, polymorphic, disordered, and anharmonic equilibrium structure. However, this allows for a variety of external stimuli, including temperature, pressure, electrostatic fields, and ultrashort light pulses to readily phase‐reconfigure WTe_2_ and, as a result, its ferroelectric and topological properties.^[^ ^6^, ^11^, ^12^, ^13^, ^15^ ^]^

Although tuning strategies to exploit the reconfigurability afforded by 2D layered materials have advanced significantly in the past decade, current device‐integrable approaches remain limited by their small tuning range and inability to act deeper inside the material (i.e., to effect a bulk phase transition).^[^ ^12^, ^16^, ^17^, ^18^ ^]^ Beyond the context of energy storage technologies, electrochemical ion intercalation offers an alternative route to overcome these limitations and to extend the space of experimentally accessible metastable states.^[^ ^7^, ^19^, ^20^, ^21^, ^22^, ^23^, ^24^ ^]^ For example, electrochemical lithium‐ion intercalation of WTe_2_ (at levels past 0.425 lithium ions per WTe_2_ formula unit) induces a structural transformation to the symmetrically distinct T_d_’ phase, which exhibits anomalously large in‐plane strains and could enable the realization of a novel type of strain actuator at the nanoscale (Figure 1a).^[^ ^25^, ^26^ ^]^ To explore this new type of phase control and to understand the mechanistic pathways that lead to such emergent dynamical properties within metastable phases, electrochemical intercalation platforms, already being explored by a variety of in situ approaches, need to be further integrated with pump‐probe techniques.^[^ ^27^, ^28^, ^29^, ^30^, ^31^ ^]^ However, thus far such a combination of electrochemical intercalation and dynamical pump‐probe approaches remains unrealized.

Here, we report the first combination of an electrochemical device with a variety of non‐contact in situ ultrafast probes, including both electron diffraction and nonlinear optical spectroscopy approaches. We demonstrate the versatility of our platform by studying two different dynamic lattice behaviors in WTe_2_, at low and high lithium‐ion loadings. Our custom‐fabricated electrochemical platform features a target WTe_2_ sample (the working electrode), which is connected to a lithium‐ion source (the counter‐electrode) via an ion‐conducting polymer electrolyte compatible with ultra‐high vacuum, as well as an external potentiostat to control the applied bias (Figure 1b). In addition, an electron‐transparent Si_3_N_4_ window under the sample allows for electron diffraction experiments to be performed in transmission mode, while a microfabricated serpentine heater near the polymer electrolyte promotes ionic conductivity for electrochemical device operation without an external temperature control. In situ ultrafast electron diffraction (UED), polarization‐ and time‐resolved second harmonic generation (SHG), and optical microscopy experiments on single‐crystalline WTe_2_ electrodes unveil new possibilities for tuning of the interlayer shear mode response and the stabilization of the T_d_ phase with increasing amounts of lithium ions.

## 2 Results and Discussion

### 2.1 Ultrafast Electron Diffraction Measurements

**Figure 2:**
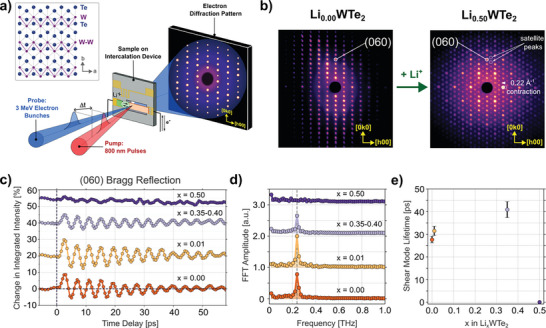
In situ UED experiments of WTe_2_ electrodes. a) Schematic representation of the experimental operando ultrafast electron diffraction setup. The inset shows the atomic arrangement of the T_d_ phase projected onto the *ab*‐plane and W‐W zigzag chains oriented along the *a*‐axis are highlighted in purple. b) Electron diffraction patterns of a single‐crystalline WTe_2_ electrode before photoexcitation in its un‐intercalated and fully lithiated states at room temperature. c) Time‐dependent diffraction behavior of the (060) Bragg peak after photoexcitation with increasing concentration of intercalated lithium ions. d) FFT of the time traces plotted in panel (c). e) 0.24 THz shear mode lifetime extracted from an exponentially decaying sine fit to curves in panel (c).

**Figure** **2**a shows a schematic representation of the pump‐probe UED setup that was operated at the SLAC National Accelerator Laboratory to characterize the photoexcited structural dynamics of WTe_2_ electrodes.^[^ ^32^ ^]^ For these measurements, 2D electron diffraction patterns of single‐crystalline WTe_2_ samples were recorded in transmission using 3 MeV ultra‐relativistic electron bunches (10 fC per bunch). The ultrafast structural response in the electrochemically lithiated samples was initiated using 800 nm pump pulses from a Ti:sapphire amplifier (100 fs duration, fluence ≈1 mJ cm^−2^), which were synchronized to the electron probe pulses and delayed in time using a mechanical delay stage.

A representative electron diffraction pattern captured on the 2D detector prior to photoexcitation of a 60 nm‐thick single‐crystalline WTe_2_ specimen is shown to the left in Figure 2b. Here, the characteristic orthorhombic pattern that is expected of the non‐centrosymmetric T_d_ phase of WTe_2_ is displayed over a large *q*‐range, consistent with prior results.^[^ ^6^, ^25^, ^33^ ^]^ The W‐W zigzag chains (i.e., *a*‐axis) are oriented horizontally, indicating that the direction of facile ion transport in WTe_2_ is positioned parallel to the applied electrochemical field.^[^ ^25^ ^]^ Upon full electrochemical lithiation, a second diffraction image of the same sample to the right in Figure 2b shows a contraction of Bragg peak positions associated with the parent orthorhombic phase, as well as the appearance of secondary reflections (i.e., satellite peaks). This new arrangement verifies the formation of an enlarged monoclinic unit cell after the phase transition, corresponding to the emergence of the lithium‐rich T_d_’ phase (see Figure 1a), consistent with previous work.^[^ ^25^, ^26^ ^]^ Notably, the retention of well‐defined Bragg peaks shows that the intercalated sample remains single‐crystalline, and the considerable *q*‐shifts in the diffraction peak positions signify the presence of large in‐plane strains along the *a*‐axis (Figure S1, Supporting Information).^[^ ^25^ ^]^ Remarkably, this intercalation‐induced phase transition is largely reversible (Figure S2, Supporting Information).

We analyze the in situ ultrafast response of the WTe_2_ lattice following 800 nm photoexcitation at various stages of electrochemical intercalation using the integrated diffraction intensity of over 200 Bragg reflections. Figure 2c showcases the sinusoidal oscillations that are inherent to this material for the intensity of the (060) peak, as an example of this analysis. A fast Fourier transform (FFT) of the signal reveals a single component at 0.24 THz (Figure 2d; Figure S3, Supporting Information) consistent with previous results.^[^ ^6^, ^33^, ^34^ ^]^ Fitting the time‐dependent diffraction signal *I(Δt)* with an exponentially decaying sinusoid of the form:

(1) $$I\left(\Delta t\right)=A\cdot e^{-\frac{\Delta t}{\tau }}\cdot sin\left(2\pi f\cdot \Delta t+\varphi \right)$$

with amplitude *A*, phonon mode decay time *τ*, frequency *f* = 0.24 THz, and phase *φ* gives an intrinsic mode lifetime of 27.6 ps (Figure 2e). A similar oscillatory signature is prevalent in the measured time‐dependent responses of other peaks across the diffraction pattern (Figure S4, Supporting Information). Specifically, the sign of the oscillation amplitude switches between positive and negative, as the order of the *k* index in (*hk0*) diffraction peaks alternates between even and odd, respectively. These findings signify the excitation of the interlayer shear mode of WTe_2_, which shifts the stacking configuration along the crystalline *b*‐axis, following the phase transition path from the non‐centrosymmetric, orthorhombic T_d_ phase toward the centrosymmetric, monoclinic 1T’ structure.^[^ ^6^, ^33^ ^]^

Turning on electrochemical intercalation and increasing the lithium content of the WTe_2_ electrode to Li_0.01_WTe_2_ (well below the concentration needed for a transition to T_d_’) surprisingly enhances the periodic modulation of the (060) peak intensity in the photoexcited state (Figure 2c). The FFT shows an increase in the shear mode amplitude by as much as 16.2%, and the decaying sine fit indicates an enhancement in the mode lifetime by 13.8% (Figure 2d). The absence of measurable *q*‐shifts or significant changes in the intensity of Bragg reflections at this small lithium‐ion content indicates that the increased modulation amplitude is not associated with any long‐range strains or a phase transition in the crystal structure (Figure S1, Supporting Information). Furthermore, no contribution from increased phonon‐ion or phonon‐electron scattering induced by ion intercalation is observed, which would be expected to decrease the lifetime and amplitude of the shear mode.^[^ ^34^, ^35^, ^36^ ^]^ Instead, the enhancement can be attributed to a concomitant rise in electron doping of WTe_2_ that acts to charge‐compensate the positive lithium ions and re‐establish charge neutrality in the system. According to prior theory and experiment, electron doping in WTe_2_ stabilizes the non‐centrosymmetric T_d_ phase relative to the centrosymmetric 1T’ structure.^[^ ^10^, ^12^ ^]^ By virtue of the nearly degenerate energies of the two phases and the small barriers separating them, a T_d_–1T’ fluctuating phase mixture is possible at room temperature.^[^ ^10^, ^12^, ^13^ ^]^ We note that we cannot distinguish between a pure phase mixture and a structure consisting of large anharmonic distortions (e.g., significant population of the saddle point region between the two phases). However, since both cases are purely associated with a sliding of the stacked layers, the structural difference between the two is only a change of the sliding amplitude. For either case, the addition of ≈10^20^ cm^−3^ electronic carriers can quench distorted 1T’‐like regions and grow the T_d_ phase fraction. Since the shear mode response is associated with a displacement toward 1T’ that is unique to the T_d_ phase and disappears in 1T’ (as observed by negligible shear mode excitation in 1T’ MoTe_2_, see ref. [6]), the T_d_ homogenization leads to a stronger shear mode signature as observed experimentally (Figure 2c; Figure S4, Supporting Information). Similarly, the reduction of anharmonic corrections resulting from the stabilization of T_d_ would increase the measured lifetime of the shear mode as observed, determined by a dephasing time reflecting the spread of excited frequencies. Separately, processes related to anharmonic scattering of the shear mode may also play a role.

This trend in electronically driven shear mode enhancement eventually reverses when the WTe_2_ electrode is further lithiated near the T_d_ to T_d_’ phase transition. For example, at a lithiation state in the range Li_0.35_WTe_2_ to Li_0.40_WTe_2_, the shear mode amplitude exhibits a significant reduction (Figure 2c), which FFT analysis quantifies as a 55% drop in the amplitude of the 0.24 THz component with respect to the pure (un‐lithiated) state (Figure 2d). At even higher lithiation levels above the T_d_’ transition (i.e., Li_0.5_WTe_2_), the shear mode signature vanishes entirely from all recorded Bragg reflections (Figure 2c; Figure S4, Supporting Information). Thus, the ability to coherently drive the shear mode is lost after the structural phase transformation to T_d_’ in the electrode. We note the retention of well‐defined diffraction peaks in this phase indicates that the disappearance of the dynamical response does not originate from a poly‐crystalline environment (Figure 2b). Rather, we interpret this as arising from the significant structural rearrangements and changes in electronic band structure associated with the T_d_’ phase, involving a larger effective distance between the two phases (see Figure 1a), larger barriers to surmount, and the suppression of the sensitivity to photodoping that the T_d_ and 1T’ phases exhibit. Thus, electrochemical lithium‐ion intercalation of WTe_2_ allows for the induction and tuning of two separate symmetry regimes inside the same specimen by accessing different electronic and structural driving mechanisms.

### 2.2 Optical Microscopy and Second‐Harmonic Generation Measurements

**Figure 3:**
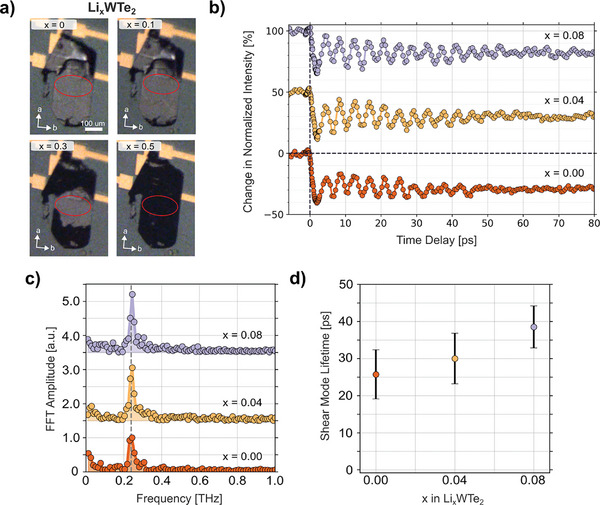
In situ optical microscopy and pump‐probe SHG experiments of WTe_2_ electrodes. a) Optical micrographs of a single‐crystalline WTe_2_ electrode during electrochemical intercalation. As lithiation proceeds, the dark color at the edges of the sample propagates inward along the *a*‐axis, which is likely associated with ripple deformations arising from the T_d_ to T_d_’ phase transformation. The red ellipse indicates the approximate position of the 800 nm probe beam. b) Time‐resolved SHG intensity of the 90° lobe maximum as a function of increasing lithiation. c) FFT of the time traces in panel (b). d) 0.24 THz mode lifetime as determined from the log fit to the extracted peaks in each time trace in panel (b).

Single‐crystalline specimens of electrochemically lithiated WTe_2_ were subjected to additional, simultaneous in situ optical microscopy and polarization‐resolved, pump‐probe SHG measurements to further elucidate the role of electronic doping and ion intercalation on the structure and dynamical response. For these experiments, the solid‐state electrolyte of the intercalation device was replaced with an optically transmissive liquid mixture that enhances the electrochemical ion insertion kinetics at room temperature.^[^ ^37^, ^38^, ^39^ ^]^ In addition, the liquid electrolyte was kept flowing to ensure a uniform chemical profile over the WTe_2_ sample.^[^ ^25^ ^]^ Optical micrographs of the WTe_2_ electrode show a metallic‐gray specimen submersed in the transparent electrolyte that has its crystallographic *a*‐axis oriented parallel to the applied electrochemical field (**Figure** **3**a). At low amounts of lithiation (i.e., Li_0.08_WTe_2_) the corners of the electrode darken. Subsequent images taken at higher stages of lithiation show an optically dark front preferentially propagating along the *a*‐axis direction, a process that continues until the sample has been fully transformed (i.e., appears completely black). This darkening of the sample is likely a consequence of the substantial rippling and buckling induced by the T_d_ to T_d_’ structural phase transition, as evidenced by SEM imaging (Figure S5, Supporting Information).

Concomitant changes in crystallographic symmetry unfolding during electrochemical intercalation were simultaneously monitored via optical pump‐probe SHG measurements by coupling 800 nm probe pulses and 2.1 µm pump pulses (to separate and filter the pump from the 800 nm probe) into the electrochemical cell. The SHG signal from a single region on the electrode was collected in reflection geometry and decomposed into a fixed polarizer basis before detection. We first verified that the shear mode followed a similar response as described above for the UED experiments. Figure 3b displays the measured pump‐probe SHG response as a function of intercalation with the probe polarization parallel to crystallographic *b*‐axis, showing sensitivity to the interlayer shear mode studied above with UED and consistent with prior results.^[^ ^6^ ^]^ Similar changes in photoexcited shear phonon amplitude and damping rates were observed as shown in Figure 3b–d, with the mode amplitude and lifetime increasing with intercalation at values below the phase transition threshold. Upon raising the lithium‐ion content of the electrode to Li_0.08_WTe_2_ to homogenize the T_d_ phase, the shear mode amplitude increases by 23%, and its decay time lengthens by 21%.

**Figure 4:**
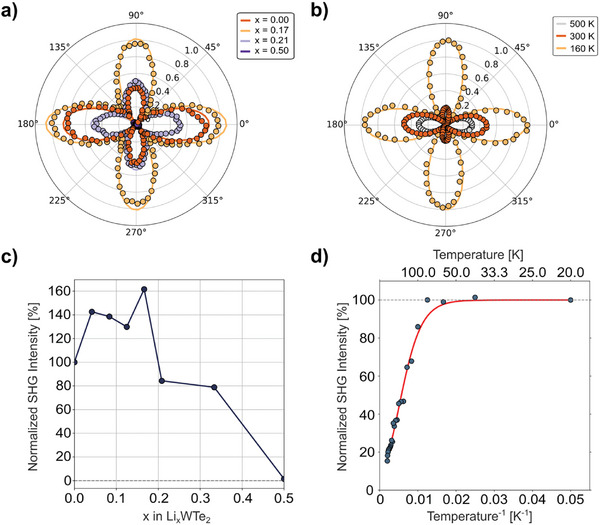
In situ SHG experiments of lithiated and pristine WTe_2_ electrodes. a) SHG polarimetry at room temperature, as a function of lithium intercalation. b) SHG polarimetry of un‐intercalated WTe_2_, as a function of temperature. c) Integrated intensity under the SHG polar curve at various stages of electrochemical lithiation of the same specimen shown in panel (a). d) Integrated intensity under the SHG polar curve at various temperatures of the pristine WTe_2_ sample shown in panel (b), and the corresponding fit using a bistable potential well model (described in main text) showing stabilization of T_d_ with decreasing temperature.

The polarization dependence of the SHG pattern provides further information on the symmetry of the structure and its evolution as a function of doping and intercalation. **Figure** **4**a,b shows the SHG polar patterns of the sample recorded with the s‐out‐channel of the analyzer. Prior to intercalation we observe a predominantly two‐lobed shape oriented parallel to the *a*‐axis, consistent with the crystallographic point group of the T_d_ phase (i.e., *mm2*).^[^ ^10^, ^12^, ^40^ ^]^ Upon lithiation of the electrode to just a few percent, the SHG intensity grows significantly and becomes more four‐fold symmetric without the presence of appreciable pattern rotations. At the same time, concurrent optical micrographs show that the dark color at the edges of the WTe_2_ electrode has begun to move into the interior of the sample, following a well‐defined front oriented perpendicular to the crystallographic *a*‐axis direction, driven by the lithium intercalation process (Figure 3a). Given that that this color change remains a few tens of µm distant from the sample region located under the laser probe, the observed increase in SHG intensity and the change in pattern symmetry do not originate from the T_d_–T_d_’ phase change and further support the above described electronically driven homogenization of the T_d_ phase at low levels of lithiation. At even higher lithium‐ion loadings (i.e., Li_0.21_WTe_2_) the SHG polar pattern continues to retain its symmetric four‐fold shape, but its intensity is significantly reduced.

To further verify the above interpretation, temperature‐dependent SHG polarimetry measurements of un‐intercalated WTe_2_ electrodes were carried out to assess the T_d_/1T’ phase competition. Specifically, the evolution of the temperature‐dependent SHG intensity provides a first experimental estimate of the energy scale separating the T_d_ and 1T’ phases. Accordingly, additional SHG measurements were performed on single‐crystalline WTe_2_ specimens cooled inside a continuous‐flow nitrogen cryostat down to 20 K. We find, as expected, that cooling leads to dramatic increases in the SHG intensity, consistent with the stabilization of the non‐centrosymmetric T_d_ phase at low temperature relative to centrosymmetric 1T’.^[^ ^41^, ^42^ ^]^ The recorded polar patterns through the s‐out channel of the analyzer reveal a familiar shape change with decreasing temperature similar to the intercalation effect: a mostly two‐lobed shape at room temperature evolves into a larger four‐fold polar pattern at 160 K (Figure 4b). To estimate the energy scale separating T_d_ and 1T’, the temperature‐dependent integrated SHG intensity (Figure 4d) was fitted using a bistable potential well model (see Methods; Figure S6, Supporting Information), which assumes the free energy landscape of WTe_2_ consists of only T_d_ and 1T’ minima separated by an energetic difference *ΔU*.^[^ ^43^ ^]^ We find:

(2) $$\frac{I_{SHG}\left(T\right)}{I_{SHG}\left(0K\right)}=\frac{1}{{\left[1+\frac{v_{2}}{v_{1}}e^{-\beta \Delta U}\right]}^{2}}$$

here, *β* = (*k_B_T*)^−1^, and *ν_1_* and *ν_2_* are the attempt frequencies for switching from 1T’ to T_d_ and vice versa. This model captures the observed temperature dependent SHG and suggests that T_d_ and 1T’ are separated by only 28.1 meV f.u.^−1^ (*ν_1_ν_2_^−1^* = 2.67). This supports the picture of random and dynamic formation of small 1T’‐like (e.g., shear distorted) domains at room temperature. To the best of our knowledge, this represents the first experimental determination of the energetic difference between the T_d_ and 1T’ phases.

### 2.3 Theory

**Figure 5:**
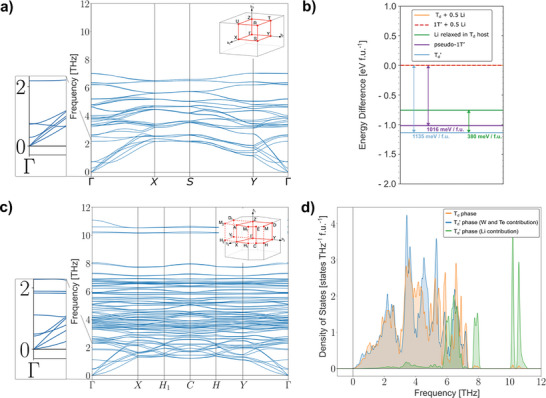
First‐principles phonon calculations and relative energetics of the T_d_, 1T’, and T_d_’ phases of WTe_2_. a) Calculated phonon dispersion of the T_d_ phase. Top inset shows the Brillouin Zone (BZ) definition, while the lower inset shows a magnification of the low‐frequency optical phonon modes. b) Comparison of the energetic difference between the possible crystallographic structures in WTe_2_. Here, the energy of half of an isolated lithium atom is included in the energies of the pristine T_d_ and 1T’ phases, such as to make the values per f.u. more comparable to those of the other lattices containing lithium ions. c) Calculated phonon dispersion of the T_d_’ phase. Top inset shows the BZ definition (see ref. [47]) and lower inset displays a zoom‐in of the low‐frequency optical modes. d) Comparison of the phonon DOS of the T_d_ and T_d_’ phases, indicating negligible changes to the low‐frequency DOS, as well as the presence of lithium‐ion‐rattling modes at much higher frequencies than the experimental frequency resolution.

The extensive tunability of the low‐frequency lattice dynamics of WTe_2_ observed above for increasing lithium‐ion concentrations calls for a deeper understanding of the phononic behavior and relative energetics of the T_d_, 1T’ and T_d_’ phases. To this end, phonon dispersions of the three structures were computed using the first‐principles DFT finite displacement method that included the PBE+D3 exchange‐correlation (xc) functional to account for interlayer van der Waals interactions (see Methods).^[^ ^44^, ^45^ ^]^ The resulting phonon spectrum computed for the pristine T_d_ phase indicates the presence of low‐frequency optical phonon modes that closely match prior experimental values (**Figure** **5**a; Table S2, Supporting Information).^[^ ^6^, ^33^, ^34^, ^46^ ^]^ Most notably, the 0.29 THz mode is associated with a small, relative displacement between the bottom and top layers acting along the *b*‐axis (i.e., the T_d_ to 1T’ transition pathway; Figure S7a, Supporting Information), and thus corresponds to the 0.24 THz shear‐mode signature observed in the MeV‐UED and SHG experiments. A similar low‐energy mode and nearly identical phonon density‐of‐states (DOS) are predicted for the metastable 1T’ phase (Figure S7b,c and Table S2, Supporting Information). However, since the laser pump photo‐dopes the material with holes which unidirectionally stabilizes the 1T’ structure over T_d_, a negligible low‐frequency modulation of the structure‐factor and second‐harmonic are expected from the 1T’ lattice.^[^ ^6^, ^10^, ^33^, ^34^ ^]^ Static lattice energy calculations of the relaxed T_d_ and 1T’ structures indicate the energetic difference between these two phases is vanishingly small (< 2 meV f.u.^−1^), which further corroborates the existence of a dynamical phase heterogeneity at room temperature and supports the observed temperature‐dependent behavior of the SHG intensity (Figure 5b).

Moreover, ab initio DFT calculations suggest that the fully‐lithiated T_d_’ phase also hosts low‐frequency optical phonon modes akin to T_d_, which are slightly stiffer and anticipated at 0.43, 0.49, and 1.12 THz (Figure 5c; Figure S7d and Table S2, Supporting Information). The addition of lithium ions does not appear to significantly change the low‐energy DOS of T_d_‐WTe_2_, but rather introduces isolated intercalant rattling modes at much higher frequencies between 7.5 and 11 THz (Figure 5d). From this perspective, the inserted lithium ions only slightly perturb the low‐frequency T_d_ phonons. However, the lack of any experimental signature of these modes in the MeV‐UED data suggests that ion‐intercalation must play an additional role in T_d_’ that causes minimal coupling between the pump and these lattice dynamics. DFT static lattice energy calculations show that the T_d_’ phase has a significantly lower energy than the relaxed T_d_ or 1T’ structures (Figure 5b) and is even more energetically distant to these phases than the static T_d_ structure with lithium atoms relaxed inside of it or the T_d_ structure relaxed simultaneously with intercalated lithium atoms (i.e., pseudo 1T’). This substantial energetic difference between T_d_’ and T_d_/1T’ is the direct result of dramatic structural distortions induced by the high lithium‐ion concentrations.^[^ ^25^, ^26^ ^]^ Constructing an alternative, non‐primitive unit cell description of T_d_ that more closely resembles T_d_’ and better visualizes any structural changes between these two lattices (Figure S7e, Supporting Information), reveals that stacked layers shift by an additional 1.8 Å relative to each other along the orthorhombic *a‐*axis (i.e., perpendicular to the T_d_ to 1T’ transformation pathway). This *a*‐axis layer shear is 4.5 times larger than the one that modulates the T_d_ to 1T’ phase change and coincides with the direction of the 0.73 THz mode of the T_d_ lattice (Figure S7f, Supporting Information).^[^ ^6^ ^]^ Consequently, not only would a multi‐phonon excitation mechanism be necessary to optically phase‐change T_d_’, but also a significant amount of energy would be needed to overcome the considerable energetic barrier to nearby phases (Figure 5b). This is consistent with the absence of any coherent signature of T_d_’ shear modes observed in MeV‐UED. Hence, whereas in the T_d_ phase, photoexcitation or hole doping leads to a stabilization of the 1T’ and thus activation of the low frequency shear mode, no such mechanism exists in T_d_’.

## 3 Conclusion

In summary, this study demonstrates the first integration between an active electrochemical device and several in situ ultrafast probes, revealing two different regimes of dynamic tunability and emergent functionality in WTe_2_: at low levels of lithiation, the electronically driven homogenization of the T_d_ phase, inducing order from a fluctuating mixture of two nearly degenerate phases, and amplifying the ultrafast lattice response of the crystal. In contrast, at high lithium‐ion concentrations, the lattice transformation to the T_d_’ structure modifies electron‐phonon coupling to the low frequency interlayer shear modes and quenches the low frequency ultrafast signature. Together, these results highlight the potential of electrochemistry to significantly modulate 2D layered material functionality, tune disorder, and potentially stabilize the novel topological and ferroelectric properties of WTe_2_. The compatibility of the on‐chip platform presented in this work could be further extended to other advanced experimental characterization techniques, such as transmission electron microscopy, which could enable further insights into the structural and dynamical behavior of materials during intercalation.

## 4 Experimental Section

### Device Fabrication for UED Experiments

Electrochemical intercalation devices for electron diffraction measurements were made following similar procedures to that described in ref. [25] Devices were fabricated in the cleanroom from commercial 300‐µm thick Si wafer substrates, covered on both sides with 50 nm of LPCVD‐grown Si_3_N_4_ (Rogue Valley Microdevices). Free‐standing Si_3_N_4_ membranes were created by photolithographically writing and dry etching local regions on the back of the wafer, and then exposing the bare Si substrate to a 30% KOH wet‐etch solution. Metal contacts and resistive heaters were patterned onto the front side and deposited with e‐Beam evaporation (3 nm Ti, 47 nm Au). Processed wafers were covered in a 20 µm protective film of photoresist and cut into single chips using a diamond‐blade wafer saw (DISCO DAD3240). Each chip was cleaned with acetone, then gently rinsed with isopropyl alcohol and DI water, and finally dried under the flow of nitrogen gas.

To form the working‐electrodes of the devices (i.e., target material to be intercalated), single‐crystalline WTe_2_ samples were mechanically exfoliated from a commercial bulk crystal (HQgraphene, CVD‐grown), and thin flakes were stamped onto SiO_2_/Si substrates. Exfoliated samples with suitable lateral dimensions (>50 um) and thicknesses (<100 nm) were identified under an optical microscope, and thicknesses were verified with an atomic force microscope. Each selected sample was then transferred onto a chip in the following manner: poly(propylene carbonate) (PPC) in anisole solution (15% PPC by weight) was spin‐coated at a rate of 1500 rpm onto the SiO_2_/Si substrate, and the resulting polymeric layer was heated on a hotplate at 80 °C for 2 min to evaporate the anisole. The dried PPC film was peeled off together with the WTe_2_ sample and aligned over the metal contact of a chip's Si_3_N_4_ membrane with the aid of an optical microscope. After lowering the PPC film onto the chip and heating it to 130 °C, the polymer stamp was slowly lifted, leaving behind the WTe_2_ sample. All chips were soaked in acetone for 10 min to remove residual PPC, then gently rinsed in isopropyl alcohol and dried under the flow of nitrogen.

To synthesize the Li‐ion‐supplying counter‐electrodes of the devices, lithium iron phosphate (LFP), poly(vinylidene fluoride) (MTI Corporation) and carbon black (C65 Timical) were mixed together in a weight ratio of 74:6:20, and subsequently dissolved in N‐methyl‐2‐pyrrolidone (Acros Organics). The resulting solution was deposited onto the metal contacts opposite to the working electrodes, and chips were heated to 90 °C under vacuum to evaporate residual solvent.

To complete the assembly of the electrochemical intercalation devices, a solid‐state, polymer electrolyte was prepared inside an argon‐filled glovebox from poly(ethylene oxide) (PEO, Sigma–Aldrich, MW 600000) and lithium perchlorate (LiClO_4_, Sigma–Aldrich), mixed together in a molar ratio of 18 EO: 1 LiClO_4_, and dissolved in acetonitrile (12: 88 of solid mixture: acetonitrile, by weight).^[^ ^38^ ^]^ The solution was carefully drop‐cast between electrodes with a micromanipulator, taking care to avoid any contact to Au metal and thereby minimize undesired side‐reactions during device operation. Finally, chips were dried under vacuum for 48 h and kept in a sealed, argon‐filled environment until the start of the UED measurements.

### Device Fabrication for SHG Experiments

Electrochemical intercalation devices for SHG spectroscopy measurements were fabricated in the cleanroom from commercial 500 um‐thick Si substrates, covered in 300 nm of wet‐thermal‐grown SiO_2_ oxide (University Wafer). After metal contacts were photolithographically patterned and deposited with e‐Beam evaporation (3 nm Ti, 47 nm Au), a 15 nm‐thick conformal layer of Al_2_O_3_ was grown with ALD to passivate side‐reactions between Au metal and the liquid electrolyte during device operation. To allow for electrical connectivity to the devices’ electrodes, small regions over the metal contacts were exposed via dry etching. Processed wafers were subsequently covered in a 20 µm protective film of photoresist and cut into chips with a diamond‐blade wafer saw (DISCO DAD3240). Each chip was then rinsed with alcohol, isopropyl alcohol, and DI water, and finally dried under the flow of nitrogen gas.

The working‐electrodes for these devices were prepared by mechanically exfoliating single‐crystalline WTe_2_ samples from a commercial bulk crystal (HQgraphene, CVD‐grown), and directly transferring the flakes onto metal contacts using a standard, polydimethylsiloxane (PDMS)‐assisted technique.

Li‐ion supplying counter‐electrodes were fashioned inside an argon‐filled glovebox by depositing a piece of Li metal (Sigma–Aldrich) onto the exposed metal region opposite to a working electrode.

To complete the assembly of the devices, each chip platform was placed inside a custom‐machined Al holder and encased with an air‐tight polyether ether ketone (PEEK) cover. A liquid electrolyte synthesized from LiClO_4_ salt dissolved in polypropylene carbonate (1M LiClO_4_), was injected through a small opening in the PEEK cover until the interior cavity of the device‐case was entirely filled.

### Electrochemical Intercalation

Insertion of lithium ions into WTe_2_ electrodes was achieved with a low‐current Biologic SP‐200 potentiostat. Two different approaches were used to access the various lithiation regimes of the intercalated samples and to accurately determine their stoichiometry:

(1) For the WTe_2_ samples used in the SHG measurements, fixed amounts of current were applied to the electrochemical cell using a potentiostat and monitored the resulting current response over time (e.g., coulombic counting). In this scenario, the large flakes prepared for these experiments (e.g., with a charge capacity greater than 10 µC), as well as the passivated current collectors, minimized electron sinks (such as side reactions between electrolyte impurities or with the current collectors). This means that each electron passed and counted (as determined by the measured and integrated current vs time) could be directly associated with one lithium‐ion entering the WTe_2_ flake.
(2) For WTe_2_ samples used in UED measurements, the size of the prepared electrodes was considerably smaller than the ones used for SHG experiments (e.g., 1 nC charge capacity vs >10 µC charge capacity). Consequently, coulombic counting can no longer be regarded as a sufficiently accurate technique to monitor the sample stoichiometry during lithiation. Instead, a fixed voltage corresponding to a certain Li_x_WTe_2_ composition (based on voltage‐stoichiometry relationships measured in ref. [25]) was applied to the electrochemical cell and allowed to reach an equilibrium value before performing the electron diffraction measurements. The intercalation values reported in this section are therefore estimates. To further improve the ionic conductivity of the polymer electrolyte in the UED experiments, an additional current was supplied by a Keithley 2410 source meter to the resistive heater on the intercalation device.

It was noted that the similarity in the time‐resolved response comparing SHG experiments to UED experiments indicates these two approaches are qualitatively self‐consistent and that thermal effects in the polymer electrolyte within the UED platform are not significantly modifying the measured response.^[^ ^48^ ^]^

### UED Measurements

The ultrafast structural dynamics of WTe_2_ electrodes were monitored at various stages of electrochemical intercalation in a pump‐probe scheme, using ultra‐relativistic electron bunches from the MeV‐UED facility at the SLAC National Accelerator Laboratory.

To generate the electron probe, 800 nm pulses from a Ti:sapphire amplifier (Coherent Legend Elite Duo) were split into two arms of the optical setup. The 800 nm signal on one arm was frequency‐tripled and directed toward a Cu photocathode at a 70° angle of incidence to photoemit electrons. The ejected charges were then collected and subsequently accelerated to 3 MeV using pulsating radiofrequency fields produced by a klystron (i.e., 360 Hz repetition rate). The resulting 100‐fs long electron bunches (≈ 10 fC per pulse) were further steered onto the sample using magnetic lenses and focused down to a 100 µm spot size (FWHM). The diffracted signal from the sample was captured in transmission‐geometry with an electron‐active P‐43 phosphor screen (located 2.15 m away from the sample) and imaged with an electron‐multiplying CCD camera (Andor iXon Ultra).

To trigger the ultrafast structural dynamics in WTe_2_, vertically polarized 800 nm pulses from the second arm in the optical setup were guided toward the sample, focused down to a 330 µm × 220 µm spot size with a fluence of ≈1 mJ cm^−2^ and spatially overlapped with the electron bunches. The arrival time between pump pulses and probe bunches was further adjusted with a mechanical delay stage. All measurements were conducted at room temperature and under vacuum.

### UED Data Processing

At any given stage of lithiation, multiple diffraction images of the WTe_2_ electrode were collected at each chosen time step *Δt*. For each recorded image, the average dark‐counts from the CCD detector were computed from the electron‐inactive, phosphor‐less corners of the P‐43 screen and subsequently subtracted out from the diffraction pattern. Bragg peaks were indexed according to previously‐determined crystal structures and the temporal evolution of their intensity after photo‐excitation (at *t_0_*) was analyzed in the following manner:^[^ ^6^, ^25^ ^]^ a square region‐of‐interest (ROI) of 1.22 Å^−1^ in diameter was centered around a particular Bragg reflection and the intensity contained in the ROI was integrated to obtain $\sum_{ROI}I$. The diffracted signal was further refined by first computing an average local background ${\bar{I}}_{LBG}$ from the corners of the ROI and then subtracting it from the ROI sum. The resulting value was normalized by the total diffraction intensity *I_TOT_* recorded in the image, and finally averaged over all images collected at given time step:

(3) $$\bar{I}={\bar{\left(\frac{\sum_{ROI}I-{\bar{I}}_{LBG}}{I_{TOT}}\right)}}_{\Delta t}$$

Relative changes in the intensity of a ROI were calculated by subtracting and normalizing the signal response by the average lattice behavior before photoexcitation ${\bar{I}}_{<t_{0}}$ (i.e., average over negative time delays *Δt* < 0):

(4) $$\frac{\Delta I}{I_{0}}=\frac{\bar{I}-{\bar{I}}_{<t_{0}}}{{\bar{I}}_{<t_{0}}}\;\cdot 100\%$$

To investigate possible frequency shifts in time of the photo‐excited 0.24 THz mode at any given level of lithiation, normalized time‐traces were subject to a DFFT (as implemented with the “specgram”‐function in Python, version 3.9.15). Here, a minimal tradeoff between frequency‐ and time‐resolution was found with 25 ps‐long Hann window segments that overlap 90% in time (Figure S3, Supporting Information).

### Optical SHG Measurements

The crystallographic symmetry of operando WTe_2_ electrodes was investigated using 50 fs‐duration, 800 nm pulses from a commercial Ti:sapphire oscillator (Positive Light Legend). The pulse train (0.3 mW, 1 kHz repetition rate) was directed through a motorized half‐waveplate onto the sample, impinging at angle of 45° relative to the surface normal. The backscattered SHG signal was isolated in reflection geometry using suitable bandpass filters, then was further decomposed into its *p*‐ and *s*‐components with a polarizer mounted on a fixed‐rotational mount (i.e., analyzer) and collected by a photomultiplier tube from Hamamatsu. All testing with the electrochemical intercalation device was conducted at room temperature and on quasi‐bulk samples, insensitive to effects associated with few layer samples.^[^ ^49^ ^]^ Additional measurements of the un‐intercalated WTe_2_ samples between 20 and 500 K were performed inside a continuous‐flow, liquid nitrogen cryostat.

For the pump‐probe SHG experiments, the polarization direction of the 800 nm probe was fixed parallel to the crystallographic *b*‐axis of the WTe_2_ sample. To discriminate between the probe and the pump signals, 2.1 µm pump pulses from an OPA system (Light Conversion TOPAS) were coupled into the optical setup to excite the sample with a power of 12 mW over an area of 123 µm (FWHM). The time of arrival between the pump and probe was adjusted using a mechanical delay stage.

### SHG Polarimetry Model

The nonlinear interaction between the incident laser probe and the non‐centrosymmetric crystal occurs mainly through an electric‐field induced second‐order polarization of the sample.^[^ ^40^ ^]^ Thus, it is sufficient to model the laser probe here as a monochromatic electric field of a single frequency *ω*, which is linearly polarized at an angle *θ_P_* relative to its *p*‐ and *s*‐components:

(5) $$E^{\left(\omega \right)}=\cos\left(\theta_{P}\right)\;\hat{p}+\sin\left(\theta_{P}\right)\;\hat{s}$$

Assuming this electric field propagates along a wavevector direction that is incident to the surface of the sample at an angle *θ_I_* in lab frame coordinates (where the sample surface is rotated in‐plane by an angle *α*), then the *p*‐ and *s*‐components of the field can be written using:

(6) $$\hat{p}=\sin\left(\alpha \right)\;\hat{x}-\cos\left(\alpha \right)\hat{y}$$

(7) $$\hat{s}=\cos\left(\alpha \right)\cos\left(\theta_{I}\right)\;\hat{x}+\sin\left(\alpha \right)\cos\left(\theta_{I}\right)\hat{y}+\cos\left(\theta_{I}\right)\hat{z}$$

The interaction between this electric field and the non‐centrosymmetric crystal is expected to then produce a second‐order, nonlinear polarization component *P_i_^(2ω)^* that is mediated by the second‐order optical susceptibility component *χ_ijk_*, as described by:

(8) $${P_{i}}^{\left(2\omega \right)}\propto \chi_{\mathrm{ijk}}{E_{j}}^{\left(\omega \right)}{E_{k}}^{\left(\omega \right)}$$

Specifically, for the T_d_ phase of WTe_2_ (point group *mm2*), the nonlinear optical susceptibility tensor takes on the following general form in contracted notation (i.e., *χ_ijk_* → *d_il_*):

(9) $$d^{mm2}=\left(\begin{array}{c} 0\\ 0\\ d_{31} \end{array}\begin{array}{c} 0\\ 0\\ d_{32} \end{array}\begin{array}{c} 0\\ 0\\ d_{33} \end{array}\begin{array}{c} 0\\ d_{24}\\ 0 \end{array}\begin{array}{c} d_{15}\\ 0\\ 0 \end{array}\begin{array}{c} 0\\ 0\\ 0 \end{array}\right)\;$$

As a result, for a laser probe incident at *θ_I_* = 45° and polarized parallel to the *b*‐axis of the WTe_2_ crystal (i.e., *α* = 0.5π), the resulting polarization‐dependent SHG response of WTe_2_ is given by the following expressions in the basis of the analyzer:

(10) $$I_{p}^{\left(2\omega \right)}\propto \;{\left|P^{\left(2\omega \right)}.\hat{p}\right|}^{2}=2d_{15}^{2}sin^{2}\left(2\theta_{P}\right)$$

(11) $$I_{s}^{\left(2\omega \right)}\propto \;{\left|P^{\left(2\omega \right)}.\hat{s}\right|}^{2}=\frac{1}{2}\;{\left[2d_{31}cos^{2}\left(\theta_{P}\right)+d_{eff}sin^{2}\left(\theta_{P}\right)\right]}^{2}$$

where *d_eff_* = 2*d_24_* + *d_32_* + *d_33_*.

### Bistable Potential Well Model

For un‐intercalated WTe_2_ samples, the free energy landscape of the system is assumed to consist of two potential‐well minima: *A*, which denotes the centrosymmetric 1T’ phase with energy *U_A_*, and *B* that represents the non‐centrosymmetric T_d_ phase with energy *U_B_* (Figure S6, Supporting Information). Both *A* and *B* are separated by a potential energy barrier *U* that mediates switching between the two wells. Assuming the attempt frequencies to switch from one potential well to another can be quantified by *ν_1_* and *ν_2_*, (from *A* to *B*, and *B* to *A*, respectively), then the resulting Arrhenius‐like switching rates between the two wells can be described by the following two coupled first‐order differential equations:^[^ ^43^ ^]^

(12) $$\frac{dn_{A}}{dt}=-v_{1}n_{A}e^{-\beta \left(U-U_{A}\right)}+v_{2}n_{B}e^{-\beta \left(U-U_{B}\right)}$$

(13) $$\frac{dn_{B}}{dt}=+v_{1}n_{A}e^{-\beta \left(U-U_{A}\right)}-v_{2}n_{B}e^{-\beta \left(U-U_{B}\right)}$$

where *n_A_* and *n_B_* represent the phase fractions of *A* and *B* respectively, and *β* = (*k_B_T*)^−1^. Assuming the above system of equations has solutions of the form:

(14) $$n_{i}={\tilde{n}}_{i}\;e^{\lambda t}$$

then the associated eigenvalues must be:

(15) $$\lambda_{1}=0$$

(16) $$\lambda_{2}=-e^{-\beta U}\;\left(v_{1}e^{\beta U_{A}}+v_{2}e^{\beta U_{B}}\right)$$

here, *λ_1_* signifies the equilibrium condition, whereas *λ_2_* captures the transient behavior. Therefore, the ratio between potential well populations at equilibrium is given by the expression:

(17) $$\frac{n_{A}}{n_{B}}=\frac{v_{2}}{v_{1}}\;e^{-\beta \Delta U}$$

where *ΔU* = *U_A_* – *U_B_*. Given that from the two possible phases, only T_d_ lacks a bulk center of inversion and thus is assumed to dominate the observed SHG signal, then the overall SHG intensity must follow:

(18) $$I_{SHG}\left(T\right)\propto n_{B}{\left(T\right)}^{2}$$

Furthermore, phase conservation dictates that:

(19) $$n_{A}+n_{B}=1$$

and, based on the observation that at low temperatures the T_d_ phase is stabilized over 1T’, the SHG intensity is expected to be maximal:

(20) $$n_{B}\left(T=0\;K\right)=1$$

As a result, the normalized SHG intensity as a function of temperature can be described by:

(21) $$\frac{I_{SHG}\left(T\right)}{I_{SHG}\left(0\;K\right)}=\frac{1}{{\left[1+\frac{v_{2}}{v_{1}}e^{-\beta \Delta U}\right]}^{2}}\;$$

### First‐Principles DFT Analysis

For a more detailed exposition of this work, readers are referred to the online report by *Rehn* et al.^[^ ^47^ ^]^ Briefly, structural relaxations of the T_d_, 1T’, and T_d_’ phases were performed with the *Vienna* Ab Initio *Simulation Package* (VASP) version 5.4.4 using both the projector‐augmented wave (PAW) method and the conjugate gradient method with three consecutive relaxation cycles to sufficiently converge the lattice energies.^[^ ^50^, ^51^, ^52^, ^53^, ^54^, ^55^ ^]^ For these calculations, the PBE+D3 xc‐functional was included to approximately account for van der Waals interactions, but the effect of spin‐orbit coupling (SOC) was neglected due to the high computational cost and the fact that the influence of SOC on the phonons dispersions is expected to be small.^[^ ^44^, ^45^ ^]^ The plane‐wave energy cutoff was set to 450 eV, the cutoff criterion for the electronic self‐consistent field (scf) cycle was set to 10^−5^ eV and that of the ionic relaxation was set to 10^−4^ eV. The *Accurate* precision setting was used in VASP, aspherical contributions to the density in PAW spheres were considered and Gaussian smearing was included with a width of 0.1 eV.

Phonon dispersion calculations were performed using *VASP* version 5.4.4 using the finite displacement method, as implemented in the *Phonopy* software version 2.22.^[^ ^56^, ^57^ ^]^ For this procedure, the path through the BZ for each phase was defined using the *AFLOW* convention, which effectively rotates the T_d_’ cell, such that the *a*‐axis is oriented in the out‐of‐plane direction.^[^ ^58^ ^]^ The plane‐wave energy cutoff was set to 300 eV, and to ensure a highly accurate calculation of forces, the electronic scf criterion was set to 10^−7^ eV and the projectors were computed in reciprocal space. Optimal convergence was found with a supercell size of 3 × 3× 1 (or 1 × 3× 3, in the case of T_d_’) and a k‐mesh of 8 × 6× 1 (or 1× 2× 2, in the case of T_d_’).

Direct visual comparisons between the T_d_ phase (orthorhombic primitive unit cell) and the T_d_’ phase (monoclinic primitive unit cell) were made by constructing a non‐primitive unit cell description of the T_d_ structure containing 4 f.u. with lattice constants *a* = 7.151 Å, *b* = 14.018 Å, *c* = 7.151 Å, *α* = 90°, *β* = 122°, *γ* = 90°. Here, the top layer of the T_d_ phase resides nearly in the same position as that of T_d_’ (excluding of course in‐plane lattice stretching and distortions). From this perspective, the bottom layer of T_d_ must translate along the orthorhombic *a*‐axis direction by 1.8 Å to match T_d_’. In other words, the T_d_ and T_d_’ structures not only differ in lattice expansion and in‐plane distortions, but also in the translation of one layer with respect to another along the *a*‐axis of the orthorhombic T_d_ cell. Importantly, this direction corresponds to the 0.73 THz shear mode of the T_d_ phase.

## Conflict of Interest

The authors declare no conflict of interest.

## Author Contributions

F.A.d.Q., P.K.M., A.S., A.P., M.E.Z., D.L., X.S., and A.M.L. participated in the collection of MeV‐UED data. M.H. and P.K. were responsible for the laser setup in the MeV‐UED beamtime. P.K.M. and E.J.S. performed in situ optical microscopy of intercalating WTe_2_ electrodes. E.J.S. and C.M.N. carried out temperature‐dependent SHG measurements of pristine WTe_2_ samples. F.A.d.Q., S.I., and L.D.M. led complementary X‐ray scattering characterization efforts. F.A.d.Q. analyzed the MeV‐UED, intercalation‐dependent SHG and temperature‐dependent SHG data. F.A.d.Q. and A.M.L. developed the polarization‐resolved SHG model and the bistable potential well model to extract the energetic difference between T_d_ and 1T’ phases. E.S.K. and D.A.R. performed ab initio DFT calculations. F.A.d.Q. and A.M.L. wrote the manuscript with input from all authors.

## Supporting information



Supporting Information

## Data Availability

The data that support the findings of this study are available from the corresponding author upon reasonable request.
