# Supplementary material for: Electrochemical Control of the Ultrafast Lattice Response of a Layered Semimetal
Source: Adv Sci (Weinh). 2024 Dec 16;12(6):2411344. doi: 10.1002/advs.202411344 (PMC11809322; doi:10.1002/advs.202411344)
Supplement: Supplementary file 1 — Supporting Information [file ADVS-12-2411344-s001.docx]

Supporting Information

Electrochemical Control of the Ultrafast Lattice Response of a Layered Semimetal

Felipe A. de Quesada^1,2^, Philipp K. Muscher^1,2^, Eliana S. Krakovsky^1,8^, Aditya Sood^4,5^, Andrey Poletayev^1,2,6^, Edbert J. Sie^1,2^, Clara M. Nyby^1,2^, Sara J. Irvine^1,2^, Marc E. Zajac^1,2^, Duan Luo^1,2^, Xiaozhe Shen^7^, Matthias C. Hoffmann^7^, Patrick L. Kramer^7^, Joel England^7^, Alexander Reid^7^, Stephen Weathersby^7^, Leora Dresselhaus-Marais^1,2,3^, Daniel A. Rehn^8^, William C. Chueh^1,2^, Aaron M. Lindenberg^1,2,3*^

*E-mail: [aaronl@stanford.edu](mailto:aaronl@stanford.edu)


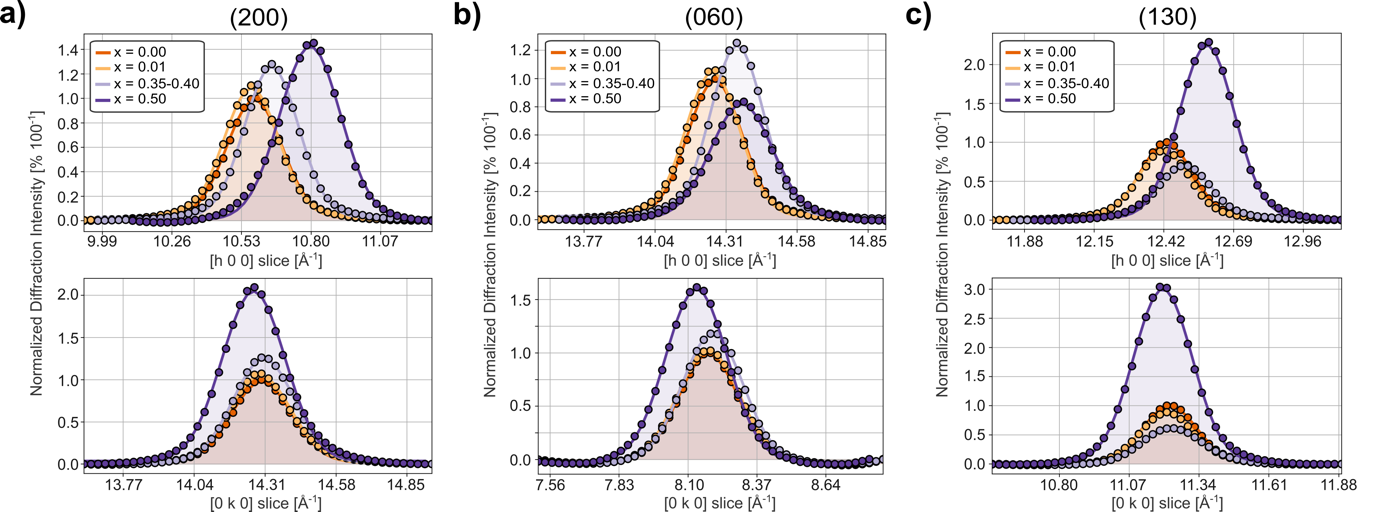


**Figure S1.** Linecuts of the normalized Bragg peak intensity along the [*h00*] and [*0k0*] directions, as a function of increasing lithiation. a) (200) peak, b) (060) peak, c) (130) peak.


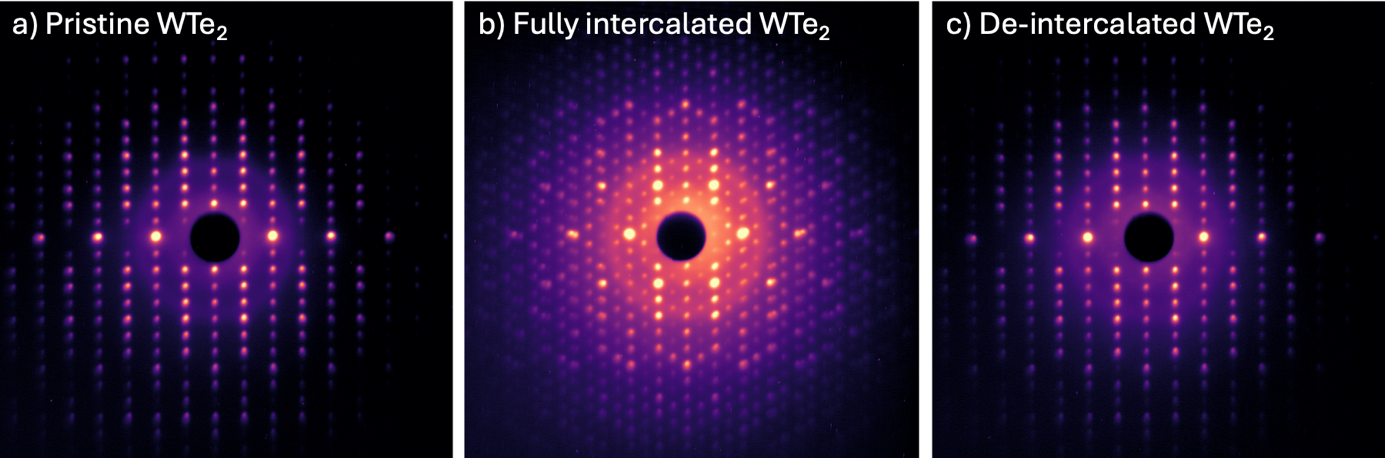


**Figure S2.** Measured electron diffraction patterns of the pristine, fully intercalated, and de-intercalated WTe_2_ structures, demonstrating the reversibility of the phase transition induced with electrochemical lithium-ion intercalation.


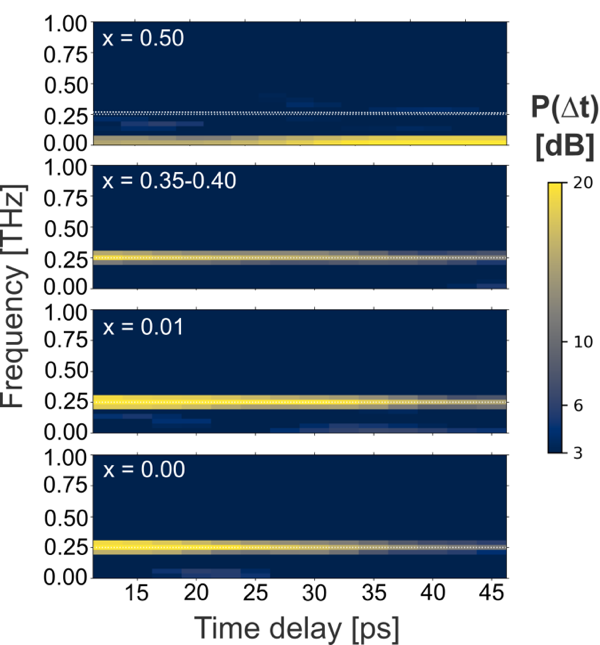


**Figure S3.** DFFT of the time-dependent (060) peak intensity in the UED diffraction pattern, as a function of increasing lithium intercalation.


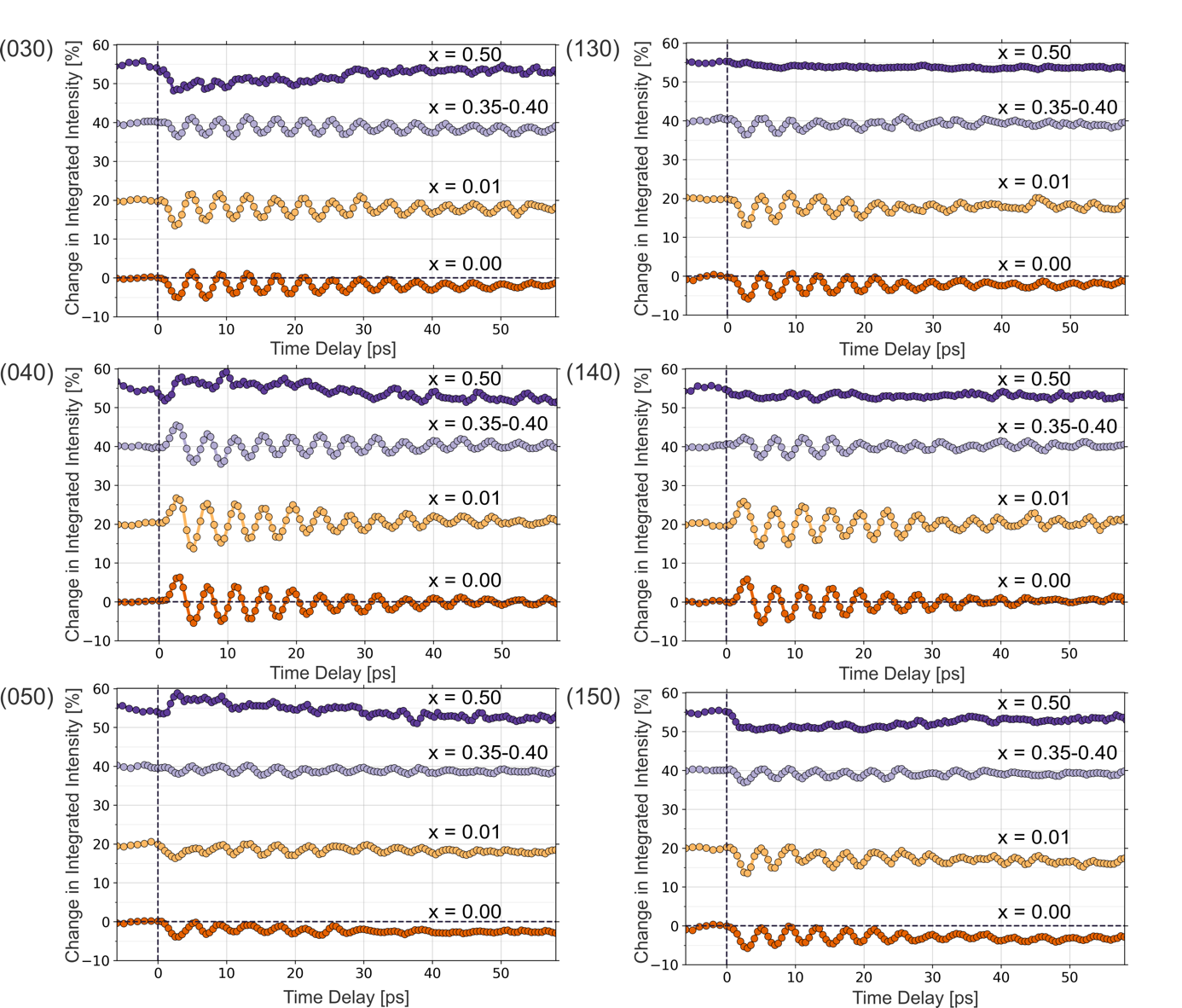


**Figure S4.** Time-dependent normalized diffraction intensity modulation after photoexcitation of various Bragg peaks, as a function of increasing lithiation.


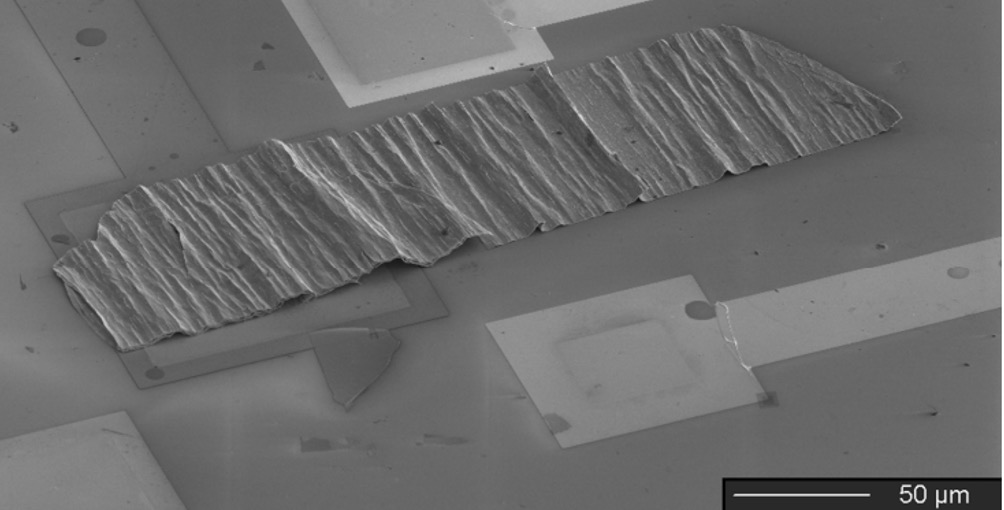


**Figure S5.** SEM image of a WTe_2_ sample after 10 cycles of lithiation and de-lithiation, showing substantial anisotropic rippling and buckling oriented perpendicular to the W-W chain direction of the crystal.


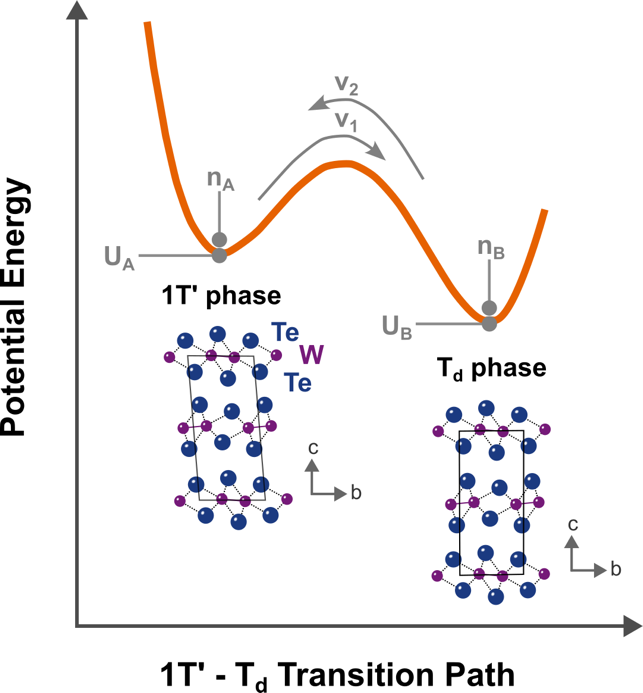


**Figure S6.** Cartoon of the bistable potential well model describing the T_d_ and 1T’ phase coexistence.


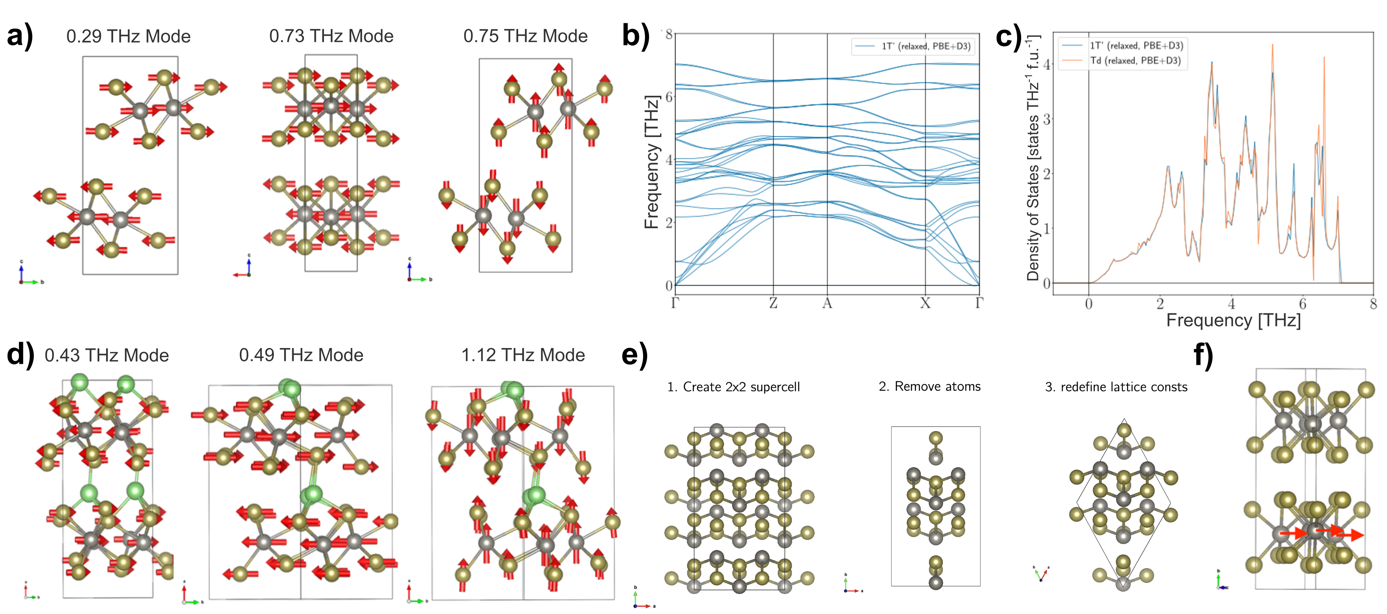


**Figure S7.** Additional first-principles DFT analysis and results. a) Visual representation of the three lowest-energy, optical modes of the T_d_ phase. b) Calculated phonon dispersion of the 1T’ phase. c) Comparison between the predicted phonon DOS of the T_d_ and 1T’ phases. d) Visual representation of the three lowest-energy, optical phonon modes of the T_d_’ phase. e) Schematic of the steps needed to construct the alternative, non-primitive unit cell for the T_d_ phase, which allows for a more direct structural comparison to T_d_’. f) Visualization of the layer displacement along the crystallographic *a*-axis that participates in the formation of the T_d_’ phase. This layer slide coincides with the direction of the 0.73 THz shear mode T_d_.

**Table S1.** Lattice parameters of each structural phase in WTe_2_ (relaxed with PBE+D3).

| **Phase / lattice parameter** | ***a* [Å]** | ***b* [Å]** | ***c* [Å]** |
| --- | --- | --- | --- |
| **T_d_** | 3.460 | 6.291 | 14.019 |
| **1T’** | 3.459 | 6.288 | 14.054 |
| **T_d_’** | 7.300 | 14.700 | 7.235 |

**Table S2.** Calculated frequency values of the three lowest-energy, optical phonons in each structural phase of WTe_2_.

| **Phase / phonon frequency** | **mode 1 [THZ]** | **mode 2 [THz]** | **mode 3 [THz]** |
| --- | --- | --- | --- |
| **T_d_** | 0.29 | 0.73 | 0.75 |
| **1T’** | 0.24 | 0.74 | 0.76 |
| **T_d_’** | 0.43 | 0.49 | 1.12 |
